# A previous champagne tap reduces the probability of traumatic lumbar puncture in the following procedure

**DOI:** 10.1038/s41598-023-46407-2

**Published:** 2023-11-10

**Authors:** Harri Sievänen, Juho Kari, Anu Huurre, Sauli Palmu

**Affiliations:** 1 Injeq Oyj, Tampere, Finland; 2 Department of Pediatrics and Adolescent Medicine, Turku University Hospital and University of Turku, Turku, Finland; 3 Department of Pediatrics, Tampere University Hospital and, Faculty of Medicine and Health Technology, Center for Child, Adolescent and Maternal Health Research, Tampere University, Tampere, Finland

**Keywords:** Biomarkers, Health care, Medical research, Oncology

## Abstract

A cerebrospinal fluid (CSF) sample containing no red blood cells (RBC), colloquially known as a champagne tap, is an ideal outcome of a lumbar puncture (LP). In this pseudoprospective study of 2573 patients aged from 0 days to 95 years, we examined in four different age categories (neonates and infants, children and adolescents, adults, and older adults) whether a champagne tap in the patient’s first LP procedure and a shorter time than 1 week between the two successive procedures are independently associated with fewer blood-contaminated CSF samples (traumatic LP) in the following procedure. One out of five CSF samples from the patient’s first LP procedures were RBC-free on average, varying from about 9% in neonates and infants to about 36% in children and adolescents. The mean incidence of champagne taps was 19.5%. According to binary logistic regression, a champagne tap in the previous LP procedure significantly determined whether the following procedure was not blood-contaminated. The odds of traumatic LP were halved or even reduced tenfold after a champagne tap. Less than a week between the two successive procedures, in turn, multiplied the odds of traumatic LP in the latter even more than tenfold. A champagne tap was not significantly associated with traumatic LP in the following procedure among pediatric patients. If the patient’s condition or therapy plan permits and the blood contamination can compromise the reliability of the CSF-based analysis and consequent diagnosis, postponing the LP procedure by several days is advisable to improve the odds of receiving a high-quality CSF sample.

## Introduction

Lumbar puncture (LP) is a clinical skill every physician should be able to perform. A cerebrospinal fluid (CSF) offers a direct means to evaluate the status of the central nervous system via pertinent laboratory analyses. However, advancing a thin spinal needle through soft tissues and bony spinous processes of the lumbar region to the spinal canal can be challenging. The sharp needle tip may damage venous plexuses located at the dorsal and ventral walls of the spinal canal and let blood cells enter the spinal canal and mix with CSF. Blood contamination of the CSF sample, called a traumatic LP (TLP), can compromise the reliability of laboratory analyses, confound a patient’s diagnosis or treatment decision, and possibly lead to unnecessary medication and prolonged hospitalization of the patient^1–3^. As CSF should not contain red blood cells (RBC), their existence in the CSF sample is a sign of abnormality, either TLP or true subarachnoid hemorrhage or other intracranial pathology. TLP is an ambiguous concept because it depends on the concentration of RBCs in the CSF sample above which level the LP procedure is considered traumatic. The criteria for TLP vary a lot between studies. In contrast, the absence of RBCs in the CSF sample is an inherently unambiguous concept and represents an optimal LP procedure, colloquially known as a champagne tap.

Champagne taps are relatively infrequent in clinical practice and their incidence seems to depend on the patient’s age^4–9^. In two retrospective studies of LP procedures done in neonates (age ≤ 28 days) and infants (age < 1 year), the reported incidences of RBC-free samples were 5% in neonates^7^, 13% in a group comprising equally neonates and infants^6^, and 26% in infants^7^. In a prospective study of pediatric LP procedures done in neonates and infants (age < 1 year) and children and adolescents (age ≥ 1 year to 18 years), the incidence of RBC-free CSF samples was 25% among the youngest patients and 30% among the older ones^4^. In a retrospective study of pediatric LPs done in children and adolescents (age < 18 years), the incidence of champagne taps was 30%, except for hemato-oncology, where the incidence was 44%^8^. In a retrospective study of LP procedures in all-aged patients, the incidence of RBC-free CSF samples was 34% in the emergency department and 24% in the rest of the hospital^5^. In another retrospective study of adult LP procedures, the incidence of champagne taps was 37% in the patient’s first LP procedure but 18% in the repeated procedure^9^.

Studies on factors contributing to a champagne tap are virtually lacking, whereas factors associated with TLP are much more investigated^4,10–21^. Success at the first attempt of LP is associated with a lower incidence of TLP in children with leukemia^10^, pediatric patients including neonates^4,11^, adults with hematologic malignancies^12,13^, and neurologic problems^14,15^. The patient’s very young age or small size, obesity-related poor visibility and palpability of lumbar bony structures, abnormal spinal anatomy, patient anxiety and movements during the procedure, coagulation disorders, physician’s inexperience in performing LP, and many attempts needed before getting a CSF sample increase the probability of TLP of occurring^5,12,16–20^. Also, a short time, from a day to 2 weeks, between successive LP procedures is known to increase the likelihood of TLP in the latter procedure^7–9,19,20^. Although the incidence of a champagne tap is not equal to one minus the incidence of TLP, presumably, the above procedural and patient-specific factors account for whether the LP procedure yields an RBC-free CSF sample.

Using pseudo-prospective design^7,9^, we examined whether a champagne tap in the patient’s first LP procedure affects the incidence of TLP in the patient’s following procedure. The advantage of pseudo-prospective design is that virtually all patient-specific confounders contributing to the success of the first LP procedure are also present in the second procedure. We hypothesized that a champagne tap in the patient’s previous LP procedure reduces the likelihood of TLP in the following one, and the short time between these two procedures has the opposite effect. We also evaluated whether the associations are different in different age categories.

## Methods

### CSF data

In this retrospective electronic health register study, we analyzed all RBC count data determined from CSF samples of all-aged patients who had undergone an LP procedure in either of two Finnish university hospitals for any reason between January 1, 2011, and May 31, 2017. The RBC count values were routinely determined from the second or third vial of the CSF sample with cytometric methods according to standard procedures of the hospital laboratories. The dataset provided for the present study was pseudonymized and comprised the RBC data, date of the LP procedure, hospital department code, and patient’s age.

The RBC count data were eligible for the present study if the patient had undergone two LP procedures in the same hospital, and the earlier was deemed the patient’s first procedure. This criterion was verified by checking that the patient had not undergone an LP procedure in 2010. One year between two successive LP procedures was considered a sufficient washout period to diminish the influence of the previous procedure on the incidence of TLP^9^. Also, a time longer than 1 year (> 365 days) between the two procedures was an exclusion criterion.

### Statistical analysis

Median and range are given as descriptive clinical data. Binary logistic regression analysis based on the likelihood ratio forward stepwise model was used as the primary statistical analysis to assess whether an RBC-free CSF sample in the patient’s first LP procedure (yes/no) or time category between the first and second LP procedure (within 1 week/a longer time) were independently associated with the incidence of TLP in the second procedure. Four common criteria for TLP were analyzed^7–9^: ≥ 10 RBCs/µL (a strict criterion employed in hemato-oncology), ≥ 500 RBCs/µL (used as an approximate criterion for visually evident blood contamination of the CSF sample), and ≥ 1000 and ≥ 10,000 RBCs/µL (both employed in neonatal and infant LP procedures).

Regression analyses were separately performed in four age categories: younger than 1 year (neonates and infants), from 1 to < 18 years (children and adolescents), from 18 to < 65 years (adults), and 65 years and older (older adults). The patient’s age at the time of the first LP procedure determined the age category. Regression analysis of the pooled data was also performed. Estimated odds ratios (OR) are reported with 95% confidence intervals (95% CI).

For descriptive purposes, cumulative distribution curves were determined to illustrate the criterion-specific incidence of TLPs in the second procedure during the 7 days after the previous first procedure. The day when at least two-thirds of all observed TLPs has occurred was considered a criterion for a meaningful reduction in the likelihood of TLP occurring.

Statistical analyses were done with IBM SPSS Statistics for Windows, version 28.0 (IBM Corp., Armonk, NY, USA). Because of four different TLP criteria and five different age categories, including the pooled data, we used a stricter threshold for the p-value to avoid spurious associations. Therefore, a p-value less than 0.0025 was considered statistically significant.

### Ethical issues

The study was performed in accordance with the Declaration of Helsinki. The regional Ethics Committee of the Expert Responsibility area of Tampere University Hospital approved the present study as a sub-study of another clinical study^22^. Since the present study was based on a selected dataset from hospital electronic health registers with no patient identifiers, the informed consent was waived by the regional Ethics Committee of the Expert Responsibility area of Tampere University Hospital.

## Results

### Data characteristics

The RBC data were available from two successive LP procedures of 2904 patients. The two procedures were performed within a year in 2573 patients (88.6% of the total potentially eligible sample), whose data were included in the present analyses. Patients’ age at the time of the first procedure ranged from 0 day to 95 years. Table 1. shows the descriptive data in the age categories for the age, the time between two successive LP procedures, and the RBC counts in these procedures. In the pooled data, the median time between the two procedures was 6 days, 61.2% (N = 1452) of the patient’s procedure was performed within a week, and 76.3% (N = 1962) of the procedures were performed within a month.

**Table 1:** Patients’ median age (range) at the time of the 1st LP procedure, median time (range) between the 1st and 2nd procedures and corresponding median RBC counts (range) broken down by age categories.

Age category	N	Age at the 1st LP (days*/years)	Time between the LPs (days)	RBC count at the 1st LP (cells/µL)	RBC count at the 2nd LP (cells/µL)
Neonates and infants	116	21 (0–361)*	7 (0–338)	267 (0–4,840,000)	204 (0–470,000)
Children and adolescents	300	7.4 (1.0–18.0)	7 (0–356)	2 (0–212,800)	6 (0–777,000)
Adults	1365	49.4 (18.1–65.0)	5 (0–364)	51 (0–4,544,000)	45 (0–1,472,000)
Older adults	792	72.2 (65.0–94.9)	5 (0–363)	86 (0–1,580,000)	79 (0–2,160,000)

### Incidence of champagne and traumatic taps

The incidence (and number) of champagne taps in the first LP procedure was 8.6% (N = 10) in neonates and infants, 36.3% (N = 109) in children and adolescents, 19.3% (N = 264) in adults, and 15.0% (N = 119) in older adults. In the pooled material, the incidence was 19.5% (N = 502).

**Figure 1:**
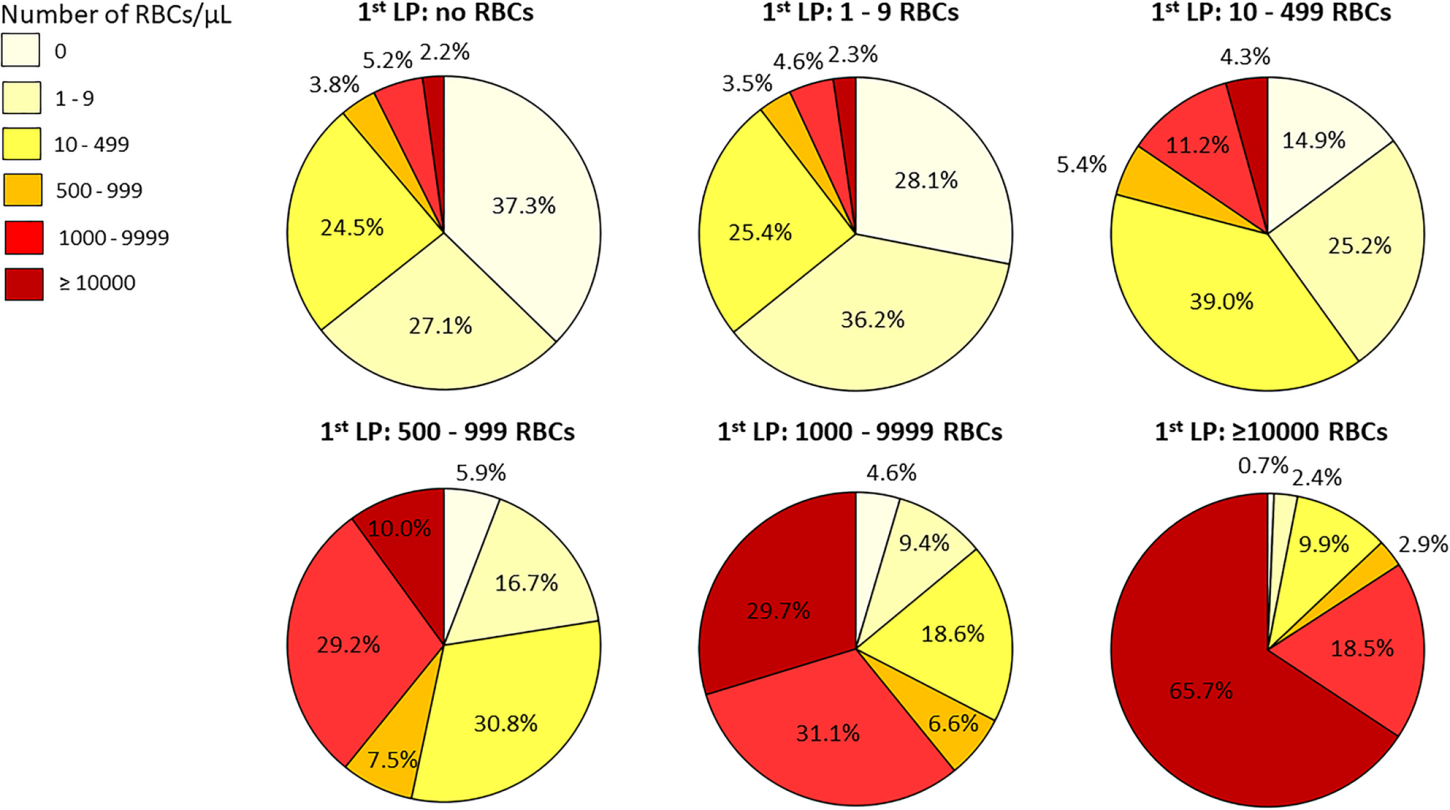
Percentage proportions of different RBC count levels in the second LP procedure in the pooled data broken down by the RBC count levels in the first procedure. The number of RBCs in the first procedure is given above the corresponding pie chart of the second procedure.

Figure 1 illustrates the proportions of different levels of RBC counts in the second LP procedure in the pooled data broken down by the RBC count levels in the first procedure. If the CSF sample in the first procedure appeared not blood-contaminated (< 500 RBCs/µL) or was RBC-free, a distinctly blood-contaminated CSF sample (≥ 10,000 RBCs/µL) in the following procedures was unlikely, occurring only in 2–4% of samples. In contrast, if the first CSF sample was distinctly blood-contaminated, two out of three CSF samples in the second LP procedure were likely to be similarly blood-contaminated.

### Determinants of traumatic second procedure in age categories

Table 2 shows independent associations of an RBC-free CSF sample in the first LP procedure and the time category (≤ 7 days vs. longer) between the successive procedures on the incidence of RBC counts ≥ 10, ≥ 500, ≥ 1000, and ≥ 10,000 RBCs/µL in the CSF sample in the second procedure in different age categories and in the pooled data.

**Table 2:** Independent associations (ORs with 95% CI) of the RBC count in the 1st LP procedure and the time between the 1st and 2nd procedures with the incidence of specified RBC count or higher in the 2nd procedure in the four age categories and pooled data.

Risk factor	The 2nd LP ≥ 10 RBCs OR (95% CI)	The 2nd LP ≥ 500 RBCs OR (95% CI)	The 2nd LP ≥ 1000 RBCs OR (95% CI)	The 2nd LP ≥ 10,000 RBCs OR (95% CI)
**Neonates and infants aged < 1 year**				
The 1st LP				
RBCs in the CSF sample	ns	ns	ns	ns
No RBCs in the CSF sample				
Time between the two LPs				
> 7 days	1	1	1	ns
≤ 7 days	21.9 (6.1–78.4)	7.3 (3.2–16.9)	5.7 (2.5–13.2)	
**Children and adolescents aged 1 to < 18 years**				
The 1st LP				
RBCs in the CSF sample	1	ns	ns	ns
No RBCs in the CSF sample	0.46 (0.28–0.75)			
Time between the two LPs				
> 7 days	1	1	1	ns
≤ 7 days	2.3 (1.4–3.7)	3.3 (1.8–6.2)	3.4 (1.8–7.9)	
**Adults aged 18 to < 65 years**				
The 1st LP				
RBCs in CSF the sample	1	1	1	1
No RBCs in the CSF sample	0.31 (0.23–0.43)	0.14 (0.08–0.22)	0.09 (0.05–0.17)	0.07 (0.03–0.20)
Time between the two LPs				
> 7 days	1	1	1	1
≤ 7 days	5.8 (4.5–7.4)	8.7 (6.5–11.7)	10.0 (7.3–13.8)	13.2 (8.0–21.6)
**Older adults aged ≥ 65 years**				
The 1st LP				
RBCs in the CSF sample	1	1	1	1
No RBCs in the CSF sample	0.41 (0.26–0.64)	0.28 (0.15–0.50)	0.17 (0.08–0.36)	0.09 (0.02–0.36)
Time between the two LPs				
> 7 days	1	1	1	1
≤ 7 days	7.8 (5.6–11.0)	11.7 (7.8–17.5)	14.0 (8.8–22.3)	18.7 (8.6–40.8)
**All-aged patients**				
The 1st LP				
RBCs in the CSF sample	1	1	1	1
No RBCs in the CSF sample	0.33 (0.26–0.41)	0.21 (0.15–0.29)	0.16 (0.11–0.23)	0.11 (0.06–0.20)
Time between the two LPs				
> 7 days	1	1	1	1
≤ 7 days	5.7 (4.6–7.0)	8.5 (6.9–10.5)	9.6 (7.6–12.2)	11.6 (8.1–16.6)

*ns* not statistically significant (p ≥ 0.0025) nor of significantly added value to the model.

In the LP procedures of neonates and infants, the RBC-free CSF sample in the first procedure was not significantly associated with the incidence of TLP in the second procedure, whereas a week or less time between the successive procedures indicated over fivefold odds of TLP (p ≤ 0.003) compared to a longer time.

In the LP procedures of children and adolescents, the RBC-free CSF sample in the first procedure was significantly associated with more than halved odds of TLP in the second procedure as per the strict criterion only (p = 0.002) compared to if the first CSF sample contained RBCs of any amount. A week or less time between two successive procedures indicated double to triple odds of TLP for all (p < 0.0001) but the most generous criterion compared to a longer time.

In the LP procedures of adults, the RBC-free CSF sample in the first procedure was significantly associated with three to more than 10 times lower odds of TLP depending on the criterion (p < 0.0001) compared to if the first CSF sample contained RBCs of any amount. A week or less time between two successive procedures indicated 6 to 13 times higher odds of TLP depending on the criterion (p < 0.0001) compared to a longer time.

In the LP procedures of older adults, the RBC-free CSF sample in the first procedure was significantly associated with over two to more than 10 times lower odds of TLP depending on the criterion (p < 0.0001) compared to if the first CSF sample contained RBCs of any amount. A week or less time between two successive procedures indicated eight to almost 20 times higher odds of TLP depending on the criterion (p < 0.0001) compared to a longer time.

In the pooled data, when the first LP procedure was RBC-free, the OR of TLP in the second procedure varied from 0.33 to 0.11 compared to that if the CSF sample in the first procedure contained RBCs of any amount (p < 0.0001). Week or less between two successive procedures indicated six to 12 times higher odds of TLP depending on the criterion (p < 0.0001) compared to a longer time. Significant ORs found in the age categories did not differ from those found in the pooled data, except those related to the time between the two successive LP procedures.

### Incidence of traumatic taps during the first post-procedural week in age categories

**Figure 2:**
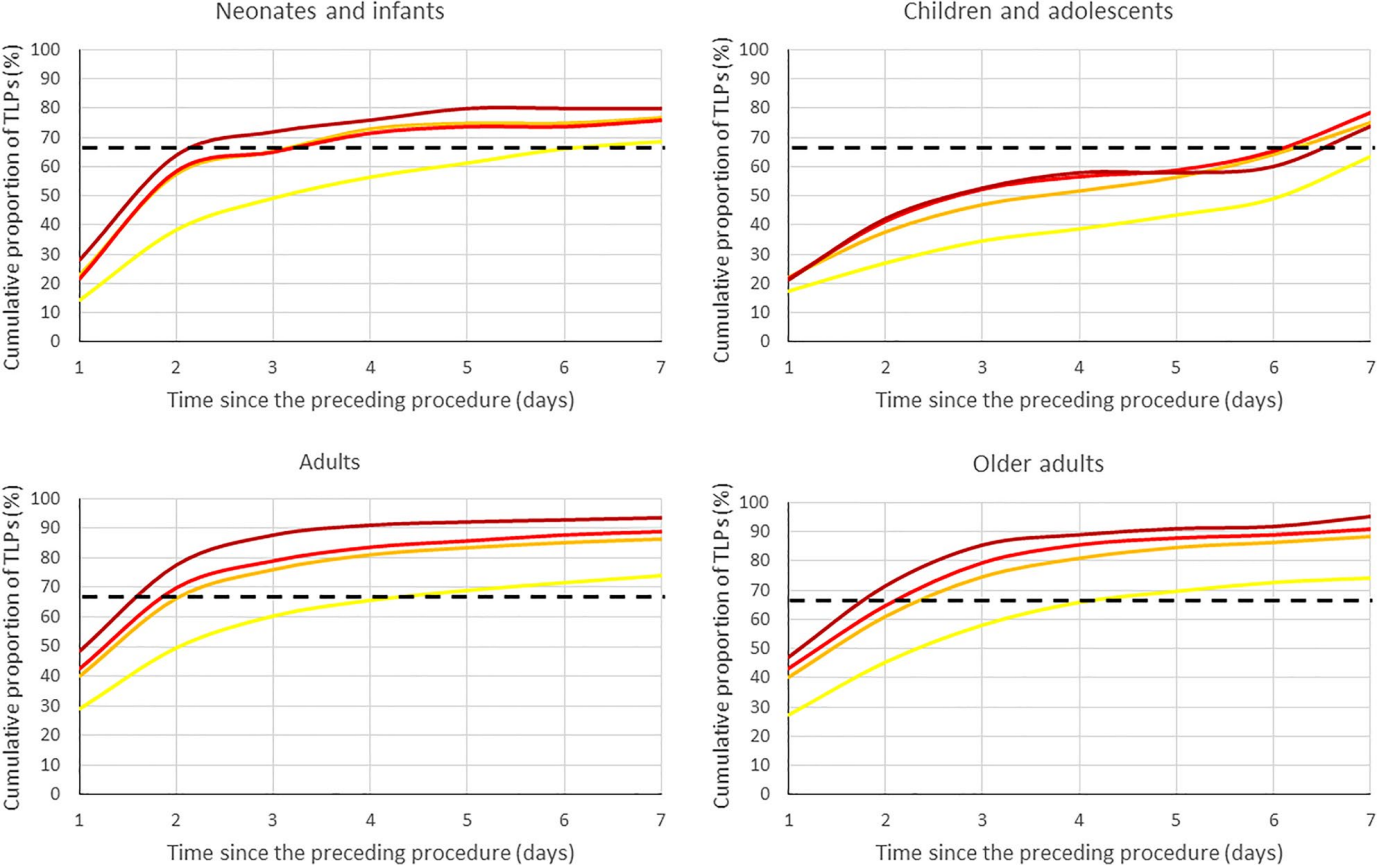
Cumulative distribution curves of TLPs during the first 7 days since the first LP procedure in different age categories according to criteria of ≥ 10 RBCs/µL (yellow line), ≥ 500 RBCs/µL (orange line), ≥ 1000 RBCs/µL (red line), and ≥ 10,000 RBCs/µL (dark red line). The dashed line indicates the level below which two-thirds of all observed TLPs have occurred.

Figure 2 illustrates the criterion-specific accumulation of TLPs during the first 7 days after the first LP procedure. Among neonates and infants using the criterion of ≥ 1000 RBCs/µL, the likelihood of TLP seems to reduce substantially in 3 days after the previous procedure. Among children and adolescents using the strict criterion of ≥ 10 RBCs/µL used in hemato-oncology, 1 week is required for substantial reduction in the likelihood of TLP. In all adults using the criterion of ≥ 500 RBCs/µL, about 2 days between the LP procedures is sufficient to substantially reduce the likelihood of TLP.

## Discussion

This study focused on the effect of the patient’s first LP procedure yielding an RBC-free CSF sample on the incidence of TLP in the following procedure. To our knowledge, this topic has not been specifically studied earlier. The present material comprised almost 2600 repeated LP procedures, ~ 60% performed within a week, among unselected all-aged patients in two university hospitals. These two hospitals provide tertiary care for about 1.8 million people in southern Finland, representing approximately one-third of the Finnish population. The results can thus be considered to represent real-life clinical data.

In clinical practice, before starting an LP procedure, it is virtually impossible to be sure that the procedure goes fluently, is successful, and provides a high-quality CSF sample—RBC-free at optimum. A champagne tap can be considered a benchmark for a technically successful LP procedure^5,6^. Within a sufficient time after a previous traumatic LP procedure or without actual cause of blood leakage into the spinal canal (e.g., a trauma-induced subarachnoid hemorrhage), the number of RBCs in the CSF sample should be zero. However, performing the LP procedure without inducing at least some blood cells into CSF is difficult^4–21^.

In the present study covering age range from neonates to over 90-year-old patients, one out of five CSF samples from the patient’s first LP procedures were RBC-free on average, the age-specific proportions being 9% in neonates and infants, 36% in children and adolescent, 19% in adults, and 15% in older adults. Corresponding data from other studies is scarce^4–6^. When the reported data on neonates and infants was pooled^4,6^, champagne taps occurred in 13% of LP procedures, whereas in children and adolescents, the respective proportion was 30%^4^, and in all-aged patients 31%^5^. The similar occurrences of RBC-free CSF samples in the present and other studies, especially among pediatric patients, may be attributed to the large number of CSF samples collected from different hospital departments (emergency, intensive care, neurology, and oncology). In adults, the lower ~ 20% incidence of champagne taps in the present study compared to the reported 31% may be due to more coherent data behind the latter value: two-thirds of CSF samples collected in the emergency department, where the providers of the LP procedure are often more experienced^5^. In older adults, challenging lumbar anatomy can complicate the LP procedure and thus reduce the odds of receiving a CSF sample without blood contamination^12,16^.

Analysis of the examined age categories revealed some notable observations. In pediatric patients, a previous champagne tap was not significantly associated with reduced incidence of TLP in the following LP procedure, in contrast with findings in adults. The low incidence of champagne taps among the youngest patients is partly explained by the relatively large diameter of the spinal needle in terms of their small-sized anatomic structures, increasing the risk of needle trauma. Two recent studies of neonates support this notion. In LP procedures done with Quincke-type needles, the incidence of TLP (defined as ≥ 500 RBCs/µL) with a 0.5 mm thick 25G spinal needle was half of that with a 0.7 mm thick 22G needle (23% vs. 47%)^23^. In another study of neonates using the same-size pencil-point needles, the incidence of TLP was 2% with the 25G needle and 23% with the 22G needle^24^. Besides the influence of needle characteristics, the rapid growth and high regeneration rate of tissues in infants likely enhance the recovery from the tissue trauma, concealing the anticipated association between the champagne tap and the incidence of TLP. Among pediatric patients older than 1 year, this association was virtually missing as well, although a plausible explanation is that about half of patients in this age category were hemato-oncology patients^8^. Their LP procedures receive high attention because of health risks caused by blood leakage into CSF^19^. Therefore, RBC-free CSF samples are significantly more frequent in hemato-oncology than in other pediatric departments (44% vs. 30%)^8^. This bimodal nature of data may have partly confounded the analysis in children and adolescents. Similar bimodality in the incidence of champagne taps seemed to exist in all-aged patients between emergency departments and other departments (34% vs. 24%)^5^. This difference was explained by the providers’ high experience and better procedural technique^5^. In the present adult LP procedures, the association between the champagne taps in the first LP procedure and the incidence of TLP in the following procedures suggested a dose–response relationship between the observed odds of TLP and the corresponding criterion of TLP in the second procedure. After a champagne tap, the more generous the criterion of TLP, the more reduced the odds of correspondingly blood-contaminated CSF sample in the following LP procedure. The odds of a distinctly blood-contaminated CSF sample were ten times lower if the CSF sample from the first LP procedure was RBC-free compared to being contaminated by blood to any extent.

Previous literature has also shown that the incidence of TLP tends to be significantly higher in the latter of two successive LP procedures, and a short time between the procedures multiplies the odds of TLP^7–9,19,20^. In line with this notion, the present study demonstrated the opposite influences of the previous champagne tap and the short time (week or less) between the successive procedures. While a champagne tap in the previous LP procedure does not preclude TLP from occurring in the following procedure, it can significantly reduce the odds of TLP occurring. However, the short time between repeated LP procedures was consistently a stronger predictor of TLP in the latter procedure.

In addition to considering a champagne tap received from the first LP procedure and the time between two successive procedures, there are several other patient-, provider-, and procedure-related factors that can contribute either positively or negatively to the success of the following LP procedure and quality of the CSF sample^4,5,12,16–20^. However, the pseudo-prospective analysis employed in the present study is presumed to control for all relevant factors affecting both LP procedures of the same patient^7,9^. Patient’s anatomy, physiology, medications, or cooperation do not essentially change, if at all, between the procedures done within a few weeks, patient’s procedures are performed in the same hospital department by similarly experienced physicians and nurses, while the size and type of the spinal needle, sedation level, patient position and other practices of the LP procedure are not changed.

Assuming that the presumptions of pseudo-prospective analysis remain valid and the present associations are correct and unbiased, the pivotal question is, what is the impact of these findings on actual clinical work? Lumbar puncture is a well-established clinical routine expected to provide reliable information about various diseases and conditions of the central nervous system and a safe means to administer intrathecal therapy. Blood contamination of the CSF sample does not mean a failed LP procedure, and many analyses of CSF used clinically are not particularly sensitive to the existence of RBCs in the CSF sample^2,25,26^. However, because of blood leakage to the spinal canal, not only RBCs but also blasts or white blood cells can mix with CSF and increase the risk of cancer in the central nervous system^19^ or complicate the diagnosis with certain consequences^3^. For example, infants with TLP (defined as ≥ 10,000 RBCs/µL) were more frequently hospitalized, although their rates of serious bacterial infections were similar to those with nontraumatic LP^1^. Regarding the potential biomarkers of Parkinson’s disease, relatively low concentrations of RBCs (50–100 RBCs/µL) can lead to the rejection of the CSF sample or otherwise compromise their analysis^27^. Blood-contaminated CSF samples may also complicate distinguishing true subarachnoid hemorrhage from TLP^28^. In sum, the benefits of optimizing the odds of receiving a CSF sample containing as few RBCs as possible are evident and worth pursuing.

While the success of the patient’s first LP procedure depends mainly on the provider (e.g., skills and experience), patient (e.g., anatomy and physiological condition), and procedural practices (e.g., the use of sedation and image guidance), it is also subject to fortuity. The odds of success in the following procedure are more determined by previously existing causes. As the present study showed, a champagne tap in the first LP procedure and at least a week between the procedures significantly increased the probability of receiving a CSF sample with a low RBC count. The pseudo-prospective analysis was presumed to control for the variance in confounding factors affecting the first procedure, but against the presumptions, some factors contributing essentially to blood-contamination of the CSF sample might have changed between the two procedures and confounded the present analysis at least to some extent. Therefore, the lack of potentially relevant data is the main limitation of the present study. Additional information on provider-, patient-, and procedure-related factors in both LP procedures might have revealed some novel associations while elucidating and contextualizing the age-specific etiologies of TLP. In this context, an intriguing relationship between TLP and lower incidence postdural puncture headache^14,29^, unestablished influence of the spinal needle type on the incidence of TLP^15,21^, and potential impact of CSF pressure^30^ call for further clinical evidence and further studies^30^.

## Conclusions

This large retrospective study based on electronic health records of CSF data showed that a champagne tap significantly accounts for whether the patient’s following LP procedure is not contaminated by blood. Depending on the criterion of TLP, the odds of a bloody CSF sample in the following procedure may reduce even tenfold if the previous procedure was RBC-free, but a distinctly blood-contaminated CSF sample is yet possible. On the other hand, less than 1 week between the consecutive LP procedures confers a strong opposite effect and multiplies the odds of TLP tenfold or more. A champagne tap was not significantly associated with traumatic LP in the following procedure among pediatric patients. If the patient’s condition or therapy plan permits and the blood contamination can compromise the reliability of the CSF-based analysis and consequent diagnosis, postponing the LP procedure by several days is advisable to improve the odds of a high-quality CSF sample.

## Data Availability

The data that support the conclusions of this study are available from Dr. H. Sievänen, upon reasonable request.
